# Clinical outcomes and aortic remodeling after Castor single-branched stent-graft implantation for type B aortic dissections involving left subclavian artery

**DOI:** 10.3389/fcvm.2024.1370908

**Published:** 2024-05-30

**Authors:** Zihui Yuan, Lihua Zhang, Fei Cai, Jian Wang

**Affiliations:** ^1^ Department of Vascular Surgery, Union Hospital, Tongji Medical College, Huazhong University of Science and Technology, Wuhan, China; ^2^ Department of Neurosurgery, Union Hospital, Tongji Medical College, Huazhong University of Science and Technology, Wuhan, China

**Keywords:** Castor single-branched stent graft, type B aortic dissection, left subclavian artery, thoracic endovascular aortic repair, aortic remodeling

## Abstract

**Background:**

The left subclavian artery (LSA) can be intentionally covered by a stent graft to acquire adequate landing zones for a proximal entry tear near the LSA during thoracic endovascular aortic repair (TEVAR). The Castor single-branched stent graft is designed to treat type B aortic dissection (TBAD) to retain the LSA during TEVAR. This study investigates clinical outcomes, aortic remodeling, and abdominal aortic perfusion patterns after TEVAR with the novel Castor device.

**Methods:**

From November 2020 to June 2023, 29 patients with TBAD involving the LSA were treated with the Castor single-branched stent graft. In-hospital clinical outcome and aortic computed tomography angiography (CTA) data were analyzed. CTA was performed preoperatively and at follow-up to observe stent morphology; branch patency; endoleak; change in true lumen (TL), false lumen (FL), and transaortic diameters; and abdominal aortic branch perfusion pattern.

**Results:**

The technical success rate was 96.6%. One failure was that the branch section did not completely enter the LSA and the main body migrated distally. No in-hospital mortality, paraplegia, or stroke occurred. During follow-up, one type Ib endoleak, four distal new entry tears, and one recurrent type A dissection arose from a new entry tear at the ascending aorta, no stent migration was observed, and the branch patency rate was 100%. At the thoracic aorta, TL diameters significantly increased, FL diameters markedly decreased, and FL was partially or completely thrombosed in most patients at follow-up. At the abdominal aorta, we observed 33.3% of TL growth and 66.7% of TL stabilization or shrinkage. The initial TL ratio at iliac bifurcation negatively predicted abdominal TL growth after TEVAR with a cutoff of 21.0%. Of the 102 abdominal aortic branches, 94.1% of the branches showed no change in perfusion pattern, 3.9% of the branches had an increased TL perfusion, and 2.0% of the branches had an increased FL contribution.

**Conclusion:**

The Castor unibody single-branched stent graft offers an efficient endovascular treatment for TBAD involving the LSA. TEVAR with the Castor device effectively induced thoracic FL thrombosis and thoracic TL enlargement and resulted in abdominal TL growth when the initial TL ratio at iliac bifurcation is less than 21.0%. Abdominal aortic branch perfusion patterns remain relatively stable after TEVAR with the Castor stent graft.

## Introduction

Type B aortic dissection (TBAD) is one of the most serious and life-threatening cardiovascular events and may have catastrophic consequences. Thoracic endovascular aortic repair (TEVAR), a less invasive technique, has rapidly become the preferred modality for TBAD, with reduced morbidity and mortality rates compared with traditional open surgical repair (1).

A sufficient proximal landing zone from 1.5 to 2.0 cm is necessary for traditional TEVAR to obtain adequate graft sealing and fixation of the stent graft (2). When proximal entry tears are located at less than 1.5 cm to the orifice of the left subclavian artery (LSA) or retrograde dissection extends to the LSA, intentional coverage of the LSA without revascularization is sometimes inevitable to obtain an excellent fixation of the aortic stent graft (3, 4). LAS blood flow may be critical for perfusion to the arm, posterior brain, and spinal cord; thus, endograft deployment in the LSA region during TEVAR carries the increased risk of posterior circulation stroke, spinal cord ischemia, and upper limb ischemia with resultant claudication (4–6). LSA revascularization is necessary in elective cases for spinal cord, upper extremity, and cerebrovascular protection. According to the retrospective analysis from a single-center experience, 23% of all the TEVAR patients required the intentional coverage of the LSA during the placement of aortic endograft, of whom 60% underwent elective LSA revascularization (7). The chimney and fenestration techniques and carotid-subclavian bypass are effective and feasible TEVAR-assistive options for preserving LSA blood flow (8). These endovascular techniques are complicated and cumbersome, increasing the risk of endoleaks and neurological complications (8). A novel commercialized single-branched TEVAR device, named Castor, could extend the sealing zone while closing the intimal tear and preserving LSA blood flow.

Aortic morphologic remodeling occurs shortly after TEVAR, and a proximal stent graft affects the downstream abdominal aortic branch perfusion. However, evaluating aortic remodeling and abdominal aortic branch perfusion pattern after TEVAR using the Castor device is rarely reported. In this study, we investigated early clinical outcomes, aortic remodeling, and abdominal aortic branch perfusion in patients with TBAD treated with LSA revascularization using the Castor single-branched stent graft.

## Patients and methods

### Ethics statement

This study complied with the principles of the Declaration of Helsinki and was approved by the institutional ethics committee of the Wuhan Union Hospital (2022IEC392). Signed informed consent was obtained from each participant who was involved in this study.

### Study population

From November 2020 to June 2023, a total of 29 patients with TBAD underwent TEVAR with the Castor unibody single-branched stent graft (MicroPort Medical, Shanghai, China) to extend the proximal landing zone. The incision criteria were as follows: (1) age from 18 to 80 years; (2) TBAD diagnosis; (3) involvement of the LSA, with the proximal landing zone potentially covering the LSA ostium; (4) distance between the proximal end of the aortic lesion and the LSA orifice, <15 mm; (5) distance between the left common carotid artery (LCCA) and the proximal end of the aortic lesion, >15 mm; (6) distance between the LCCA and the LSA orifice, >5 mm; and (7) distance from the origin of the left vertebral artery (LVA) to the LSA orifice, >25 mm. The exclusion criteria were as follows: (1) aortic congenital connective tissue disease such as Marfan syndrome; (2) Stanford A aortic dissection; (3) diameter of external iliac artery or common femoral artery, <7 mm; (4) severe stenosis and calcification of the LSA and the left brachial artery (LBA); (5) diameter of the LSA, <6 mm; (6) aberrant right subclavian artery; (7) allergy to nitinol or iodine contrast agents; and (8) previous medical history of TEVAR.

### Stent graft

The Castor single-branched stent graft is designed to treat TBAD involving the LSA and to revascularize the LSA with a branched section while sealing the entry tear. This self-expandable stent-graft endoprosthesis was made of nitinol and covered with woven Dacron polyester fabric without distal or proximal bare stents. The stent is typically deployed using a 24F exterior sheath. The diameter of the LSA at 25 mm from the ostium, the distance from the origin of the LVA to the LSA ostium, the distance from the intimal tear to the LSA ostium, the distance from the intimal tear to the LCCA ostium, the distance between the LSA ostium and the LCCA ostium, and the diameters of the proximal and distal landing zones were measured by preoperative computed tomography angiography (CTA). Four parameters were decided according to the preoperative CTA measurements: the diameter of the proximal and distal ends of the main body portion, the diameter of the distal end of the branch section, and the distance from the proximal end of the main body to the branch section. The diameters of the main body and the branch section were oversized by approximately 10%.

### Endovascular procedure

All procedures were conducted under general anesthesia in a hybrid international suite. We percutaneously inserted a 14F sheath into the right femoral artery (RFA). The LBA was exposed surgically and canulated with a short 7F sheath. A 6F sheath was inserted percutaneously into the left femoral artery (LFA), and a 5F pigtail catheter was advanced over a guidewire from the LFA into the ascending aorta, and then aortography was performed to assess visceral perfusion and determine the location of the initial intimal tears. A 4F MPA catheter was impelled into the 7F catheter along with a guidewire and taken out from the RFA. The guidewire was then withdrawn, and the MPA catheter was left as a traction conduit for the traction wire of the LSA branch section.

**Figure 1:**
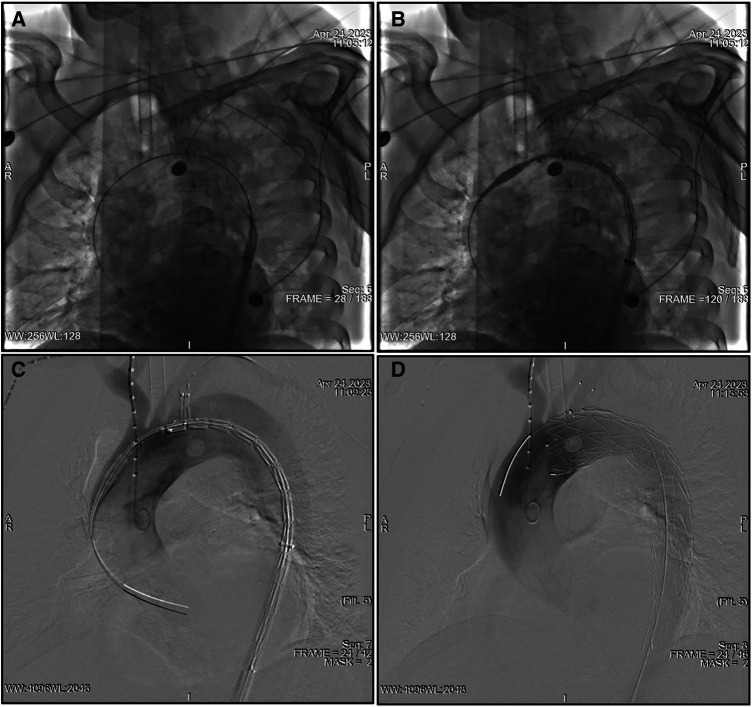
TEVAR using the Castor unibody branched stent graft for TBAD. (**A**) The traction wire of the branch section was drawn into the catheter from the RFA to the LBA, and the main body was advanced over the super stiff guidewire into the descending aorta. (**B**) The outer sheath remained in the descending aorta, and the stent graft within the soft sheath was advanced into the arch. (**C**) The branch section was drawn into the LSA by removing the soft sheath. (**D**) The main body was deployed, and, subsequently, the branch section was released by drawing the traction wire. LBA, left brachial artery; LSA, left subclavian artery; RFA, right femoral artery; TEVAR, thoracic endovascular aortic repair; TBAD, type B aortic dissection.

After the aortography was finished, a 5F pigtail catheter along with a guidewire was delivered from the RFA into the ascending aorta and then exchanged for a super stiff guidewire. The traction wire of the branched section was threaded into the MPA catheter from the RFA to the LBA, and the main body was introduced along the stiff guidewire from the RFA into the descending aorta (Figure 1A). The catheter and traction wire were continuously moved along the advancement of the main body. The delivery system was rotated until the branch was located at the greater curvature of the aortic arch. While the outer sheath of the main body stayed in the descending aorta, the stent graft within the soft inner sheath was delivered into the appropriate location in the arch (Figure 1B). The soft sheath was removed, and the branched section was pulled into the LSA by drawing the traction wire (Figure 1C). The main body was quickly released by withdrawing the trigger wire, and the branched section was subsequently deployed by withdrawing the traction wire and removing the “cap” (Figure 1D). Aortography was performed immediately to evaluate the branch patency and to reveal whether the intimal tear was well-sealed or an endoleak had occurred. Technical success was defined as the instant postoperative aortogram demonstrating complete exclusion of the entry tear, preserved LSA patency, and the absence of an endoleak. Each patient was given aspirin (100 mg/day) after the operation.

### Data collation

In-hospital outcomes were acquired from the medical records, including baseline characteristics of the patients, surgical time, length of hospital stay, and perioperative mortality. Stroke and paraplegia were recorded postoperatively. All patients underwent postoperative follow-up, and none were lost to follow-up. Aortic CTAs were performed preoperatively, postoperatively, and during follow-up. Endoleak, stent migration, branch patency, new entry, and recurrent dissection were postoperatively recorded according to CTA images.

Preoperative and the latest follow-up CTA were reviewed and compared for research analysis. Centerline analysis was performed by two experienced surgeons on a three-dimensional Leonardo™ workstation (Siemens, Munich, Germany), and the maximum diameter of the aorta orthogonal to the centerline was evaluated at the stented thoracic aorta, the thoracic aorta distal to the stent, and the abdominal aorta. True lumen (TL) and false lumen (FL) diameters were measured perpendicular to the intimal flap at the cross-sectional plane. FL status was qualitatively assessed on CTA as completely thrombosed, partially thrombosed, or patent. Abdominal branch perfusion patterns were described according to vessel lumen origin: TL, FL, both lumens (BL), and obstructed. The origins of the celiac artery (CA), superior mesenteric artery (SMA), and bilateral renal artery were recorded before TEVAR and during the most recent follow-up. Remodeling of individual branch perfusion was determined by comparing lumen origin and vessel patency for pre-TEVAR vs. follow-up. The branch perfusion pattern was defined as positive when there was an increased TL and decreased FL contribution to the blood supply. In addition, branch perfusion switching from only FL to BL was also identified as positive. Conversely, branch perfusion remodeling was characterized as negative when FL perfusion increased with the loss of TL perfusion or branches with TL or FL to the occlusion after TEVAR. TL changes in the abdominal aorta were defined as growth (>3 mm increase), stabilization (≤3 mm increase or decrease), and shrinkage (>3 mm decrease) in the TL diameter at the latest follow-up time point as compared with the measurement preoperatively. The TL ratio was calculated as the ratio of TL to transaortic diameters. Preoperatively, the initial TL ratios were measured in the following levels: proximal intimal tear, diaphragm, left renal artery (LRA), iliac bifurcation, and narrowest TL. We also calculated the number of distal entry tears and abdominal branches supplied by TL at preoperative CTA examination.

### Statistical analysis

All statistical tests were carried out using SPSS software version 20.0 (SPSS Inc., Chicago, IL, USA). For the categorical variables, the number and percentage of patients in each category were calculated and compared using Fisher's exact test. The continuous variables were expressed as mean with standard deviation for normally distributed data and as median with interquartile range for the non-normally distributed data. Univariate analysis between the two groups was performed using Student's *t*-test for the normally distributed data and the non-parametric Mann–Whitney *U*-test for the non-normally distributed data.

Binary logistic regression was used to verify multiple predictor variables and their correlation with abdominal TL growth after TEVAR. Receiver operating characteristic (ROC) curve analysis was performed to evaluate the predicting capacity of each of the parameters on TEVAR-induced TL enlargement in the abdominal aorta. ROC curves and the area under the curve (AUC) were calculated. The optimal cutoff value was determined as the point at which the Youden index was maximized by the ROC curve. Sensitivities, specificities, and prediction accuracies were then obtained with regard to the cutoff point. A *p*-value of <0.05 indicated that the difference was statistically significant.

## Results

### Baseline characteristics, dissection anatomy, and device parameters

This study included a total of 29 patients who underwent TEVAR with the Castor stent graft for TBAD. The average age was 50.2 ± 12.0 years (range, 29–66 years), and 86.2% were male. The comorbidities included hypertension (28/29, 96.6%), diabetes (3/29, 10.3%), chronic kidney disease (1/29, 3.4%), and coronary artery disease (1/29, 3.4%). In addition, the previous medical history included a prior stroke (3/29, 10.3%), alcohol use (11/29, 37.9%), and active smoking (11/29, 37.9%). Alcohol use was defined as any past and present history of alcohol. Detailed baseline demographics and medical history are summarized in Table 1.

**Table 1:** Baseline characteristics of patients, preoperative CTA measurements, and stent-graft configuration.

Variables	*n* = 29
Ages, years	50.2 ± 12.0
Male, *n* (%)	25 (86.2%)
Comorbidities, *n* (%)	
Hypertension	28 (96.6%)
Diabetes	3 (10.3%)
Chronic kidney disease, CKD	1 (3.4%)
Coronary artery disease, CAD	1 (3.4%)
Peripheral artery disease	0 (0.0%)
Chronic obstructive pulmonary disease, COPD	0 (0%)
Prior stroke	3 (10.3%)
Active smoker	11 (37.9%)
Alcohol user	11 (37.9%)
Distance from the intimal tear to the LSA ostium, mm	6.3 (2.4; 12.0)
Distance from the intimal tear to the LCCA ostium, mm	23.4 ± 8.1
Distance between the LSA ostium and the LCCA ostium, mm	12.8 (7.7; 15.2)
Distal diameter of the LSA at 25 mm from ostium, mm	9.2 ± 0.7
Distance from the origin of the LVA to the LSA ostium, mm	42.5 ± 6.1
Proximal landing zone diameter, mm	30.1 ± 3.1
Proximal stent-graft diameter, mm	32.0 (30.0; 36.0)
Oversizing rate (%)	8.8 ± 2.8
Distance from the proximal end of the main body to the branch, mm	10.0 (5.0; 10.0)
Diameter of the distal end of the branch section, mm	10.0 (10.0; 10.0)
Diameter of the distal end of the stent, mm	26.0 (24.0; 30.0)

CTA, computed tomography angiography; LSA, left subclavian artery; LCCA, left carotid common artery; LVA, left vertebral artery.

Anatomical features of TBAD and selected parameters of the stent graft according to preoperative CTA are shown in Table 1. The distance from the intimal tear to the LSA ostium was less than 15 mm in all patients, with an average of 6.3 mm (interquartile range, 2.4–12.0 mm). The distance from the intimal tear to the LCCA ostium was 23.4 ± 8.1 mm. The distance between the LSA ostium and the LCCA ostium was more than 5 mm in all patients, with an average of 12.8 mm (interquartile range, 7.7–15.2 mm). The average diameter of the LSA at 25 mm distal from the ostium was 9.2 ± 0.7 mm. The distance from the origin of the LVA to the LSA ostium was 42.5 ± 6.1 mm. The diameter of proximal landing zones was 30.1 ± 3.1 mm. The average proximal diameter of the stent graft was 32.0 (interquartile range, 30.0–36.0 mm). The oversize rate of the proximal aortic landing zone was 8.8% ± 2.8%. The average distance from the proximal end of the main body to the branch was 10 mm (interquartile range, 5.0–10.0 mm). The average diameter of the distal end of the branch section was 10 mm (interquartile range, 10.0–10.0 mm). The average diameter of the distal end of the main body was 26 mm (interquartile range, 24.0–30.0 mm).

### In-hospital and follow-up clinical outcomes

Angiographic and clinical outcomes after TEVAR are summarized in Table 2. Primary technical success was achieved in 96.6% of patients. One technique failure was that the branch section slid out of the LSA lumen and the main body migrated distally during deployment, which caused a proximal anterior type Ia endoleak. This might be attributed to the slow deployment of the main body and the simultaneous insufficient strain of the branch traction wire. When deploying the stent-graft trunk, the traction wire of the branch section was loose and not tightly pulled. The mean surgical time was 106.7 ± 38.4 min. The mean lengths of preoperative and postoperative stay in hospital were 13.9 ± 7.4 and 7.5 ± 3.1 days, respectively. One pseudoaneurysm of the LBA was observed 12 h after the operation, and open surgical repair was performed immediately. There were no deaths, strokes, paraplegia, or left upper limb ischemia within an in-hospital period.

**Table 2:** In-hospital and follow-up clinical outcomes.

Variables	*n *= 29
In-hospital outcomes	
Surgical success, *n* (%)	28 (96.6%)
Surgical time, minutes	106.7 ± 38.4
Length of stay, days	
Preoperative in-hospital, days	13.9 ± 7.4
Postoperative in-hospital, days	7.5 ± 3.1
Access route complications, *n* (%)	1 (3.4%)
Perioperative mortality, *n* (%)	0 (0%)
Paraplegia, *n* (%)	0 (0%)
New-onset stroke, *n* (%)	0 (0%)
Left upper limb ischemia, *n* (%)	0 (0%)
Follow-up outcomes	
Follow-up, months	3.0 (1.5; 10.1)
Follow-up of <12 months, *n* (%)	22 (75.9%)
Follow-up of ≥12 months, *n* (%)	7 (24.1%)
Endoleak, *n* (%)	1 (3.4%)
Proximal new entry tear, *n* (%)	1 (3.4%)
Recurrent type A dissection, *n* (%)	1 (3.4%)
Distal new entry tear, *n* (%)	4 (13.8%)
Stent migration, *n* (%)	0 (0%)
Branch patency, *n* (%)	29 (100%)

The median length of follow-up was 3.0 months (interquartile range, 1.5–10.1), and no patients were lost to follow-up. Of the 29 patients, 22 (75.9%) had a follow-up of less than 12 months, and 7 (24.1%) had equal to or more than 12 months. One type Ib endoleak from the distal stent-graft attachment site was found during the 27-month follow-up. The distal endoleak was subtle and sealed spontaneously within 12 months. One new entry tear was located at the proximal sinotubular junction at 12 months postoperatively, and recurrent type A dissection developed. A total aortic arch replacement was successfully performed, and postoperative recovery was uneventful. Four patients showed new entry tears at the abdominal aorta distal to the stent graft. Stent migration and branch occlusion were not found, and branch patency was 100%.

### Aortic remodeling: average aortic diameters and FL thrombosis

**Figure 2:**
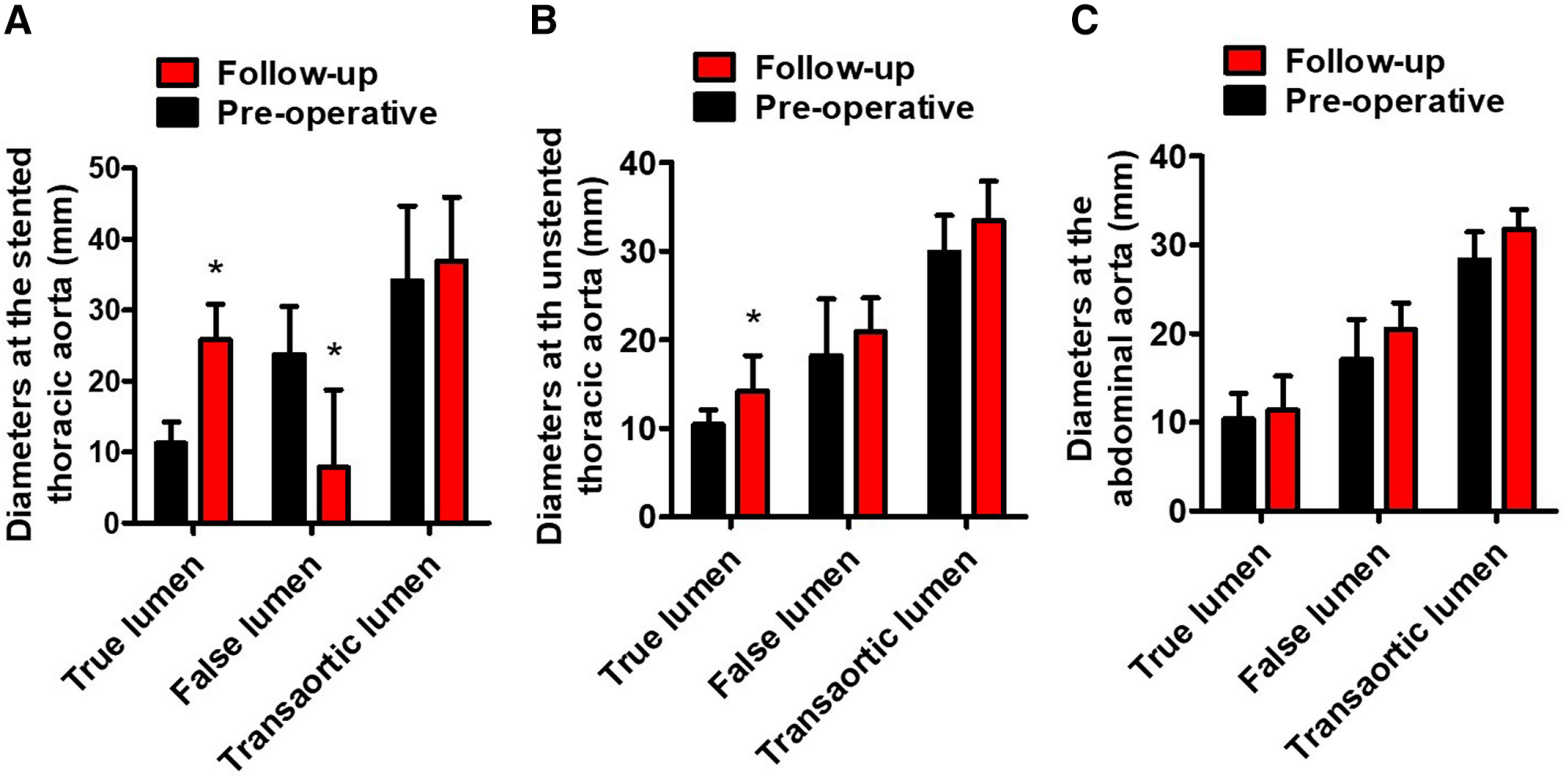
Average TL, FL, and transaortic diameters preoperatively and at follow-up. (**A**) The average TL diameters of the stented thoracic aorta increased, the average FL diameters of the stented thoracic aorta decreased, and the average transaortic diameters of the stented thoracic aorta did not change. (**B**) The average TL diameters of the unstented thoracic aorta increased. (**C**) The average TL, FL, and transaortic diameters of the abdominal aorta did not differ statistically. TL, true lumen; FL, false lumen. *P < 0.05 vs. preoperative.

Table 3 lists the average TL, FL, and transaortic diameters in three aortic segments preoperatively and at follow-up. From pre-procedure to follow-up, a significant increase in average TL diameters, a significant decrease in average FL diameters, and no change in average transaortic diameters were observed in the stented thoracic aorta. At the unstented thoracic aorta, average TL diameters expanded significantly, average FL diameters remained relatively stable, and average transaortic diameters increased slightly. At the abdominal aorta, average TL, FL, and transaortic diameters slightly increased, but these differences did not reach the statistical difference. Average TL, FL, and transaortic diameters at the stented thoracic aorta (A), unstented thoracic aorta (B), and abdominal aorta (C) were compared between pre-procedure and follow-up in Figure 2. Table 3 lists the LSA diameters distal to the branch stent preoperatively and at follow-up. There were no significant differences in the vessel diameters of unstented covering between preoperation and follow-up.

**Table 3:** Average aortic diameters and false lumen status in different aortic segments as well as LSA diameters preoperatively and at the latest follow-up.

Variables	Preoperative	Follow-up	*P-*value
The stented thoracic aorta, mm			
True lumen	11.2 (8.0; 14.3)	26.6 ± 5.8	<0.001
False lumen	23.8 (19.3; 30.5)	7.9 (0.0; 18.8)	<0.001
Transaortic lumen	34.2 (32.4; 44.7)	36.9 (31.8;45.9)	0.708
The unstented thoracic aorta, mm			
True lumen	10.5 (8.6; 12.1)	14.2 (11.3; 18.2)	0.001
False lumen	19.7 ± 9.5	20.9 (13.3; 24.7)	0.972
Transaortic lumen	29.9 (26.1; 34.1)	33.5 (29.0; 37.9)	0.116
The abdominal aorta, mm			
True lumen, mm	10.4 (7.2; 13.3)	11.3 (9.6; 15.2)	0.147
False lumen, mm	16.9 ± 9.3	19.2 ± 8.9	0.330
Transaortic lumen	28.3 (25.2; 31.4)	32.0 ± 6.0	0.069
LSA diameters distal to the stent, mm	8.9 ± 1.2	10.2 ± 0.9	<0.001
The stented thoracic aorta, *n* (%)			
Patent	22 (75.9%)	0 (0%)	<0.001
Partial thrombosis	7 (24.1%)	3 (10.3%)	
Complete thrombosis (obliteration)	0 (0)	26 (89.7%)	
The unstented thoracic aorta, *n* (%)			
Patent	21 (72.5%)	3 (10.3%)	<0.001
Partial thrombosis	5 (17.2%)	16 (55.2%)	
Complete thrombosis (obliteration)	3 (10.3%)	10 (34.5%)	
The abdominal aorta, *n* (%)			
Patent	25 (86.3%)	16 (55.2%)	0.007
Partial thrombosis	1 (3.4%)	10 (34.5%)	
Compete thrombosis (obliteration)	3 (10.3%)	3 (10.3%)	

LSA, left subclavian artery.

**Figure 3:**
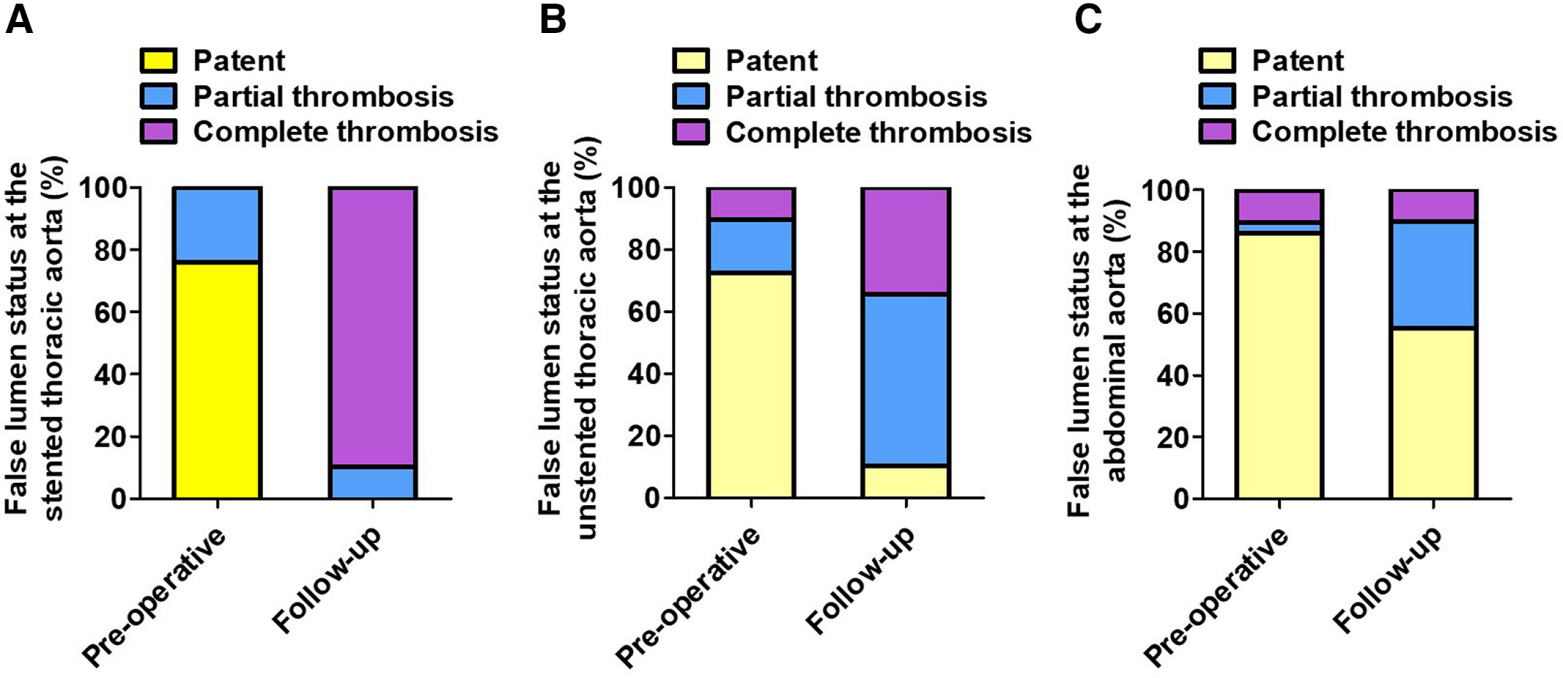
The FL status at different aortic segments preoperatively and at follow-up. (**A**) The stented thoracic FL was completely thrombosed in the majority of patients at follow-up. (**B**) The unstented thoracic FL achieved partially or completely thrombosed in most patients following TEVAR. (**C**) TEVAR induced partial thrombosis of abdominal FL in approximately 30% of patients. FL, false lumen; TEVAR, thoracic endovascular aortic repair.

Table 3 lists the FL status of three aortic segments preoperatively and at follow-up. At the stented thoracic aorta, 75.9% of FL was patent, and 24.1% of FL was partially thrombosed preoperatively, whereas 89.7% of FL was completely thrombosed and 10.3% of FL was partially thrombosed at follow-up. At the unstented thoracic aorta, patent FL decreased significantly from 72.5% preoperatively to 10.3% at follow-up, and partially and completely thrombosed FL increased markedly from 27.5% preoperatively to 89.7% at follow-up. At the abdominal aorta, patent FL decreased from 86.3% preoperatively to 55.2% at follow-up, and partially thrombosed FL increased from 3.4% preoperatively to 34.5% at follow-up. The status of FL from pre-procedure to follow-up at the stented thoracic aorta (A), unstented thoracic aorta (B), and abdominal aorta (C) is shown in Figure 3.

### Aortic remodeling: representative CTA preoperatively and at follow-up

**Figure 4:**
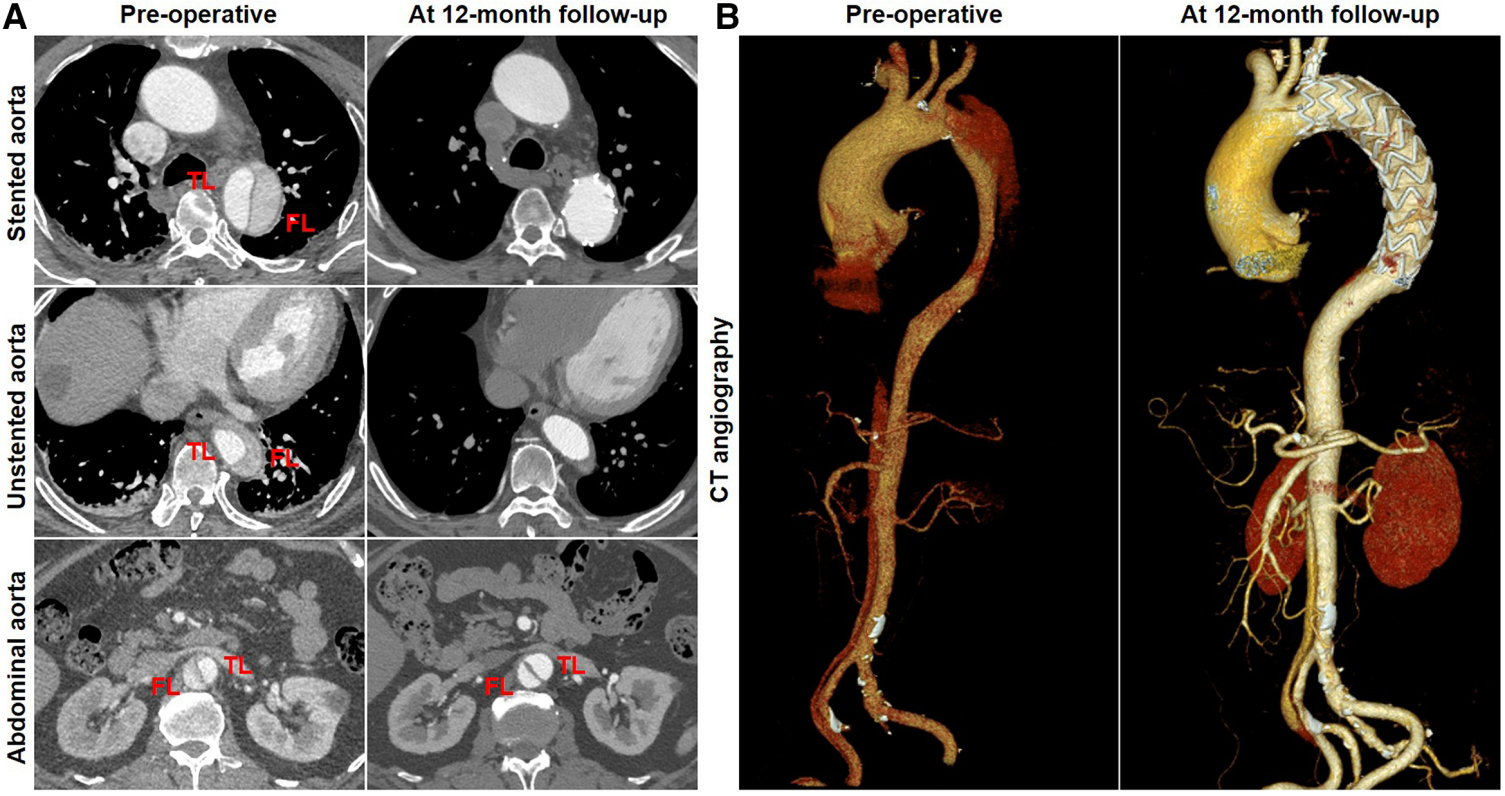
Favorable aortic remodeling after TEVAR with the Castor stent graft. (**A**) Cross-sectional CT images were obtained from three aortic segments. At the 12-month follow-up, TL enlargement, FL thrombosis and obliteration, and transaortic stabilization were observed over the stented and unstented thoracic aorta; patent FL was stable at the abdominal aorta after TEVAR. (**B**) Three-dimensional reconstructed CT images exhibited excellent remodeling at the stented and unstented aorta and stable configuration at the abdominal aorta. CT, computed tomography; FL, false lumen; TEVAR, thoracic endovascular aortic repair; TL, true lumen.

Favorable aortic remodeling was explained as FL thrombosis, FL obliteration, TL enlargement, and transaortic stabilization. A 66-year-old woman was diagnosed with TBAD, underwent TEVAR with the Castor stent graft, and completed a 12-month CT follow-up. Cross-sectional and three-dimensional reconstruction CT images are illustrated in Figures 4A,B, respectively. At 12 months following TEVAR, FL was entirely thrombosed and regressed, and TL was mostly recovered over the whole stented and unstented thoracic aorta. FL was patent but did not enlarge, and TL was relatively stabilized over the abdominal aorta 12 months after TEVAR.

### Abdominal aortic remodeling: TL changes and predictors

Changes in TL diameters at the abdominal aorta were further categorized into growth (an increase of >3 mm), shrinkage (a decrease of >3 mm), or no change (an increase or a decrease of 3 mm). In the overall patients, 72.4% (21/29) of patients had TBAD involving iliac bifurcation. Of the remaining 21 patients, 14 (21, 66.7%) had either a stable or a shrinking abdominal TL lumen, and 7 (21, 33.3%) showed an increasing TL lumen at the abdominal aorta. Table 4 summarizes the TL, FL, and transaortic diameters in two categories of abdominal aorta preoperatively and at follow-up.

**Table 4:** Abdominal aortic diameters preoperatively and at the latest follow-up.

Variables	Preoperative	Follow-up	*P-*value
True lumen growth, mm			
True lumen	2.9 (2.1; 7.9)	9.3 ± 3.1	0.017
False lumen	18.2 ± 2.9	13.3 ± 4.6	0.037
Transaortic lumen	22.4 ± 1.6	22.6 ± 2.7	0.901
True lumen shrinkage or no change, mm			
True lumen	11.6 ± 2.4	9.1 ± 0.8	0.614
False lumen	9.6 ± 3.3	17.1 ± 7.1	0.294
Transaortic lumen	21.3 ± 3.0	26.2 ± 6.7	0.259

**Figure 5:**
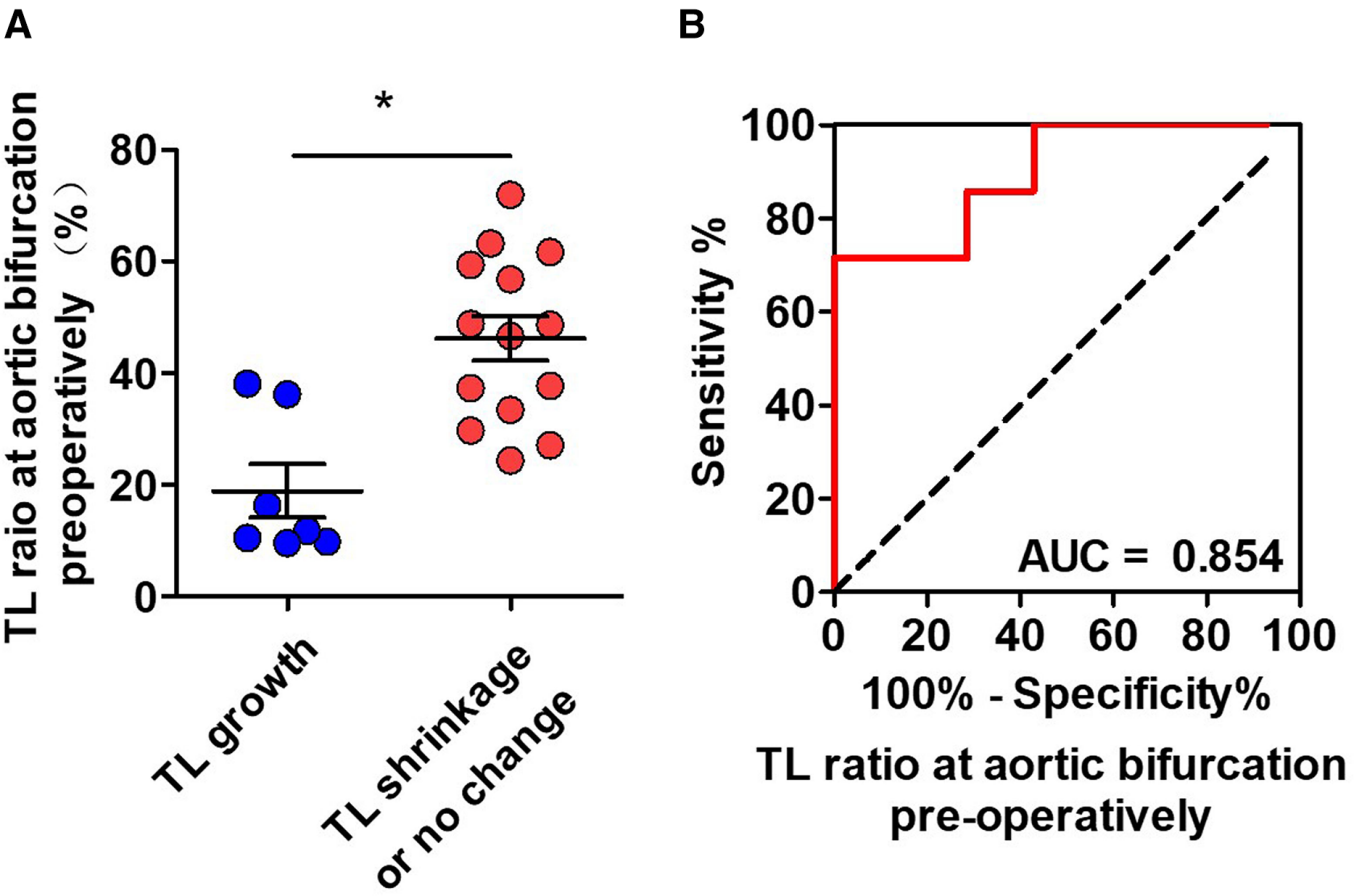
ROC analysis for the prediction of abdominal TL growth following TEVAR by the preoperative CT measurements. (**A**) The initial TL ratio at iliac bifurcation was statistically lower in patients with abdominal TL growth than in patients with abdominal TL stabilization/shrinkage. (**B**) ROC curves indicated that the initial TL ratio at iliac bifurcation was an independent predictor for abdominal TL growth after TEVAR with an AUC of 0.854. AUC, area under the curve; ROC, receiver operating characteristic; TL, true lumen; TEVAR, thoracic endovascular aortic repair. *P < 0.05 vs. TL growth.

Binary logistic regression was used to assess CT measurements that may be predictive of TL growth after TEVAR. The prediction factors included TL ratios at the level of intimal tears, diaphragm, LRA, iliac bifurcation, narrowest TL, abdominal branches affected by FL, and the number of distal tears. Candidate predictive factors for TL growth at the abdominal aorta are summarized in Table 5. The TL ratio at the narrowest TL was statistically lower in patients with abdominal TL growth than that in patients with abdominal TL stabilization or shrinkage, but was found as not a significant predictor. The TL ratio at iliac bifurcation was identified as an independent predictor of abdominal TL growth (*P *= 0.031; OR, 0.885; 95% CI, 0.793–0.989) (Figure 5A). ROC analysis was performed to evaluate the ability of the TL ratio at iliac bifurcation to predict abdominal TL growth following TEVAR. The TL ratio at iliac bifurcation had the best test performance to predict abdominal TL growth under the AUC of 0.854 (Figure 5B). A ratio of 21.0% or less was the best cutoff to predict TL augmentation, which resulted in a sensitivity of 77.8%, a specificity of 87.5%, and an accuracy of 77.8% (Figure 5B).

**Table 5:** The potential predictors measured by preoperative CT for abdominal TL remodeling.

CT measurements	TL growth	TL shrinkage or no change	*P*-value
TL ratio (%)			
Intimal tears	33.2 ± 15.0	33.8 ± 12.0	0.923
Diaphragm	26.2 ± 14.8	33.4 ± 13.8	0.281
Left renal artery	26.4 ± 19.4	36.5 ± 10.0	0.126
Aortic bifurcation	11.7 (9.8; 36.2)	46.2 ± 15.0	0.001
Narrowest TL	13.0 ± 7.4	29.4 (22.0; 32.7)	0.001
Abdominal branches by TL, *n* (%)	19 (67.9%)	37 (66.1%)	0.814
Distal entries, *n* (%)	6 (85.7%)	9 (74.3%)	0.613

TL, true lumen; OR, odd ratio; CI, confidential interval.

### Abdominal aortic remodeling: representative CTA preoperatively and at follow-up

**Figure 6:**
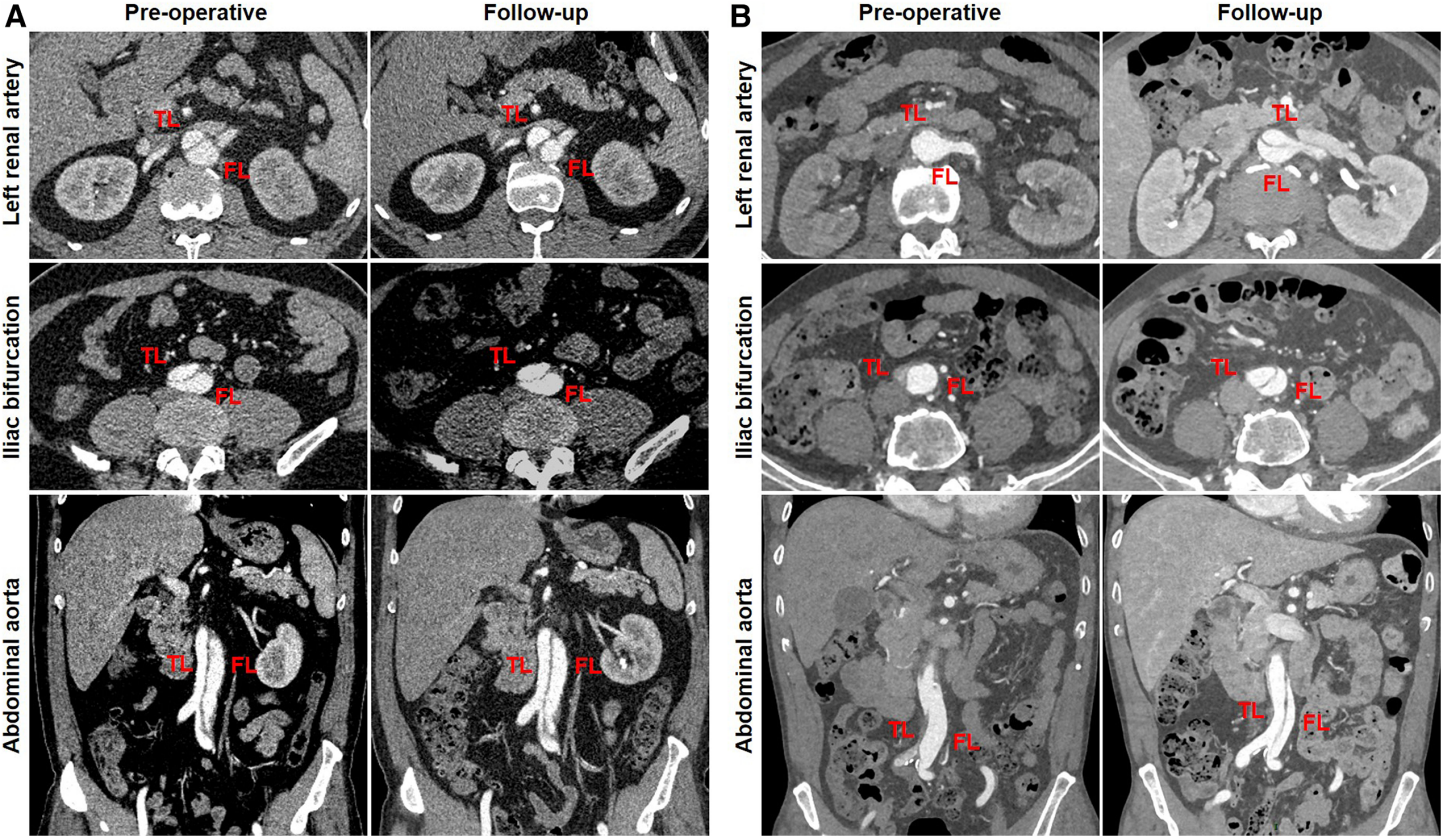
Abdominal TL growth or stabilization after TEVAR with the Castor stent graft. (**A**) The initial TL ratio at iliac bifurcation was measured as 46.6%, which exceeded a cutoff prediction value of 21.0%. Abdominal TL diameter did not alter at the 12-month follow-up. (**B**) When the initial TL ratio at iliac bifurcation was 10.5% below the cutoff value of 21.0%, abdominal TL diameter was significantly increased at 1-month follow-up. TEVAR, thoracic endovascular aortic repair; TL, true lumen.

A total of 21 patients were divided into two groups according to the changes in TL diameters at the abdominal aorta: TL growth (*n* = 7) and TL stabilization/shrinkage (*n* = 14). Representative CT images of two categories of TL growth and TL stabilization/shrinkage are shown in Figure 6A,B, respectively. CT images in the horizontal plane were illustrated at the level of LRA and iliac bifurcation, and CT images in the coronal plane were obtained at the abdominal aorta. A 52-year-old man diagnosed with TBAD had a preoperative TL ratio at iliac bifurcation of 34.6% (cutoff of >21.0%). At 12 months after TEVAR, his abdominal TL diameter was maintained stable between pre-procedure and follow-up (12.4 vs. 13.8 mm at LRA level and 10.4 vs. 10.7 mm at aortic bifurcation) (Figure 6A). A 64-year-old man diagnosed with TBAD showed a TL ratio at aortic bifurcation of 11.1% (cutoff of <21.0%). One month later, his abdominal TL diameter was markedly enlarged from pre-procedure to follow-up (2.8 vs. 10.8 mm at LRA level and 2.1 vs. 11.7 mm at aortic bifurcation) (Figure 6B).

### Abdominal aortic remodeling: branch perfusion

**Figure 7:**
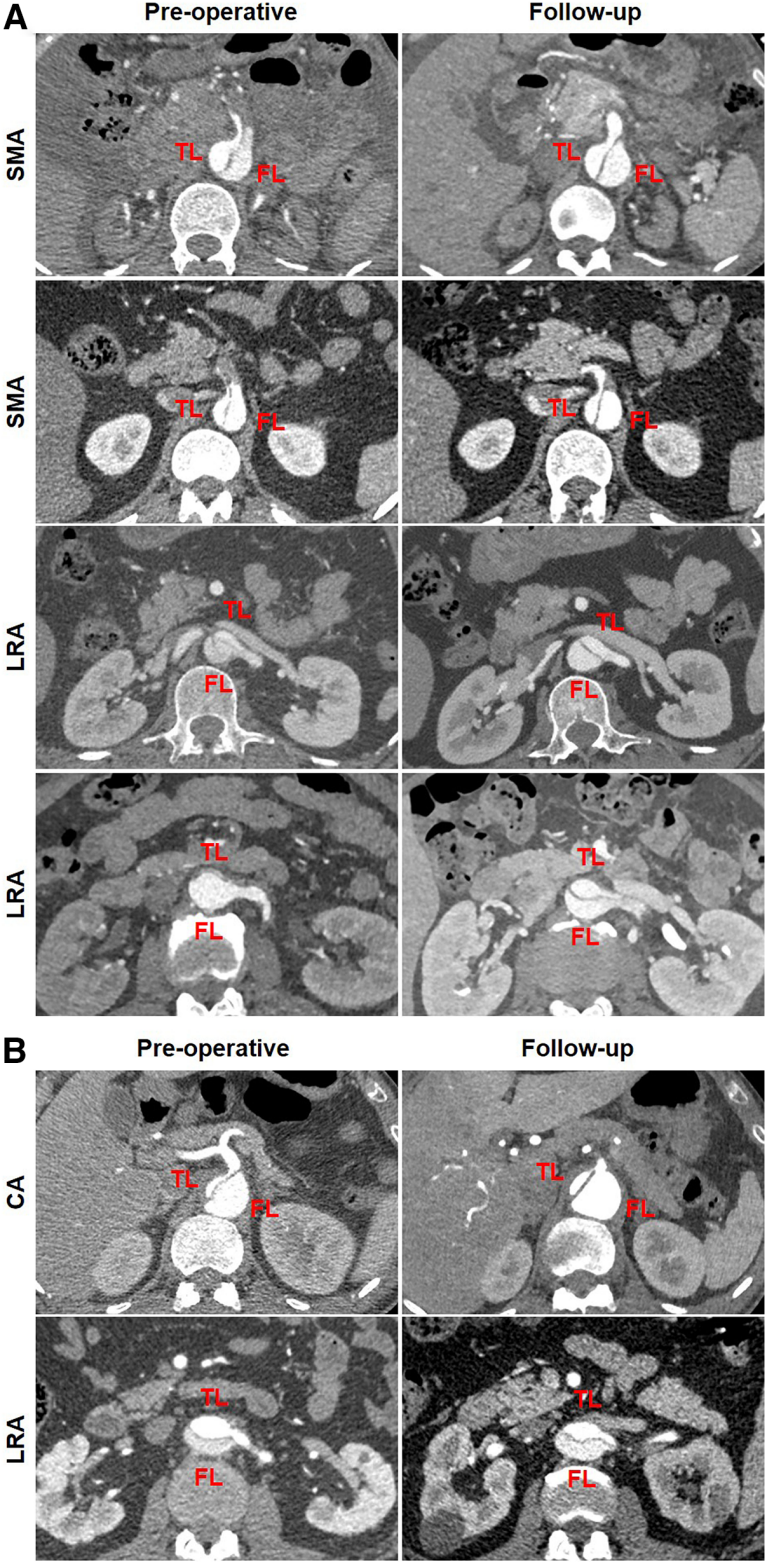
Abdominal aortic branch remodeling after TEVAR. (**A**) In the patterns of positive remodeling, the SMA was perfused by BL preoperatively and by TL at follow-up, one LRA was perfused by BL preoperatively and by TL at follow-up, and one LRA was perfused by FL preoperatively and by BL at follow-up. (**B**) In the patterns of negative remodeling, the CA was perfused by TL preoperatively and BL at follow-up, and the LRA was perfused by TL preoperatively and BL at follow-up. BL, bilateral lumen; CA, celiac artery; FL, false lumen; LRA, left renal artery; RRA, right renal artery; SMA, superior mesenteric artery; TEVAR, thoracic endovascular aortic repair; TL, true lumen.

Table 6 summarizes the abdominal aortic branch perfusion pattern preoperatively and at follow-up. Abdominal aortic branches were mostly perfused by a TL, including the CA (65.4%, 17/26), SMA (84.0%, 21/25), LRA (60.0%, 15/25), and RRA (60.0%, 15/25). Renal artery perfusion was frequently involved in dissection with 40% of the LRA or RRA by a FL or BL. Approximately 3.9% of the branches (4/102) were characterized as positive remodeling, including two SMA and two LRA. Negative remodeling was seen in 1.9% of the branches (2/102) consisting of one CA and one LRA. The majority of the branch perfusions (94.1%, 96/102) did not change after TEVAR. There were no statistical differences in TL, FL, and BL origination between pre-procedure and follow-up. Positive branch remodeling is illustrated in Figure 7A. Two SMAs were in transit from BL preoperatively to TL at follow-up. One LRA switched from BL preoperatively to TL at follow-up. One LRA was altered from FL preoperatively to BL at follow-up. Negative branch remodeling is indicated in Figure 7B. The CA changed from TL to BL after TEVAR, and LRA switched from TL to BL after TEVAR.

**Table 6:** Abdominal branch perfusion patterns preoperatively and at the latest follow-up.

Abdominal branches	Preoperative	Follow-up	*P-*value
Celiac artery, *n* (%)	26	26	1.000
True lumen	17 (65.4%)	16 (61.6%)	
Both lumens	4 (15.4%)	5 (19.2%)	
False lumen	5 (19.2%)	5 (19.2%)	
Superior mesenteric artery, *n* (%)	25	25	0.667
True lumen	21 (84.0%)	23 (92.0%)	
Both lumens	4 (16.0%)	2 (8.0%)	
False lumen	0 (0.0%)	0 (0.0%)	
Left renal artery, *n* (%)	25	25	1.000
True lumen	15 (60.0%)	15 (60.0%)	
Both lumens	2 (8.0%)	3 (12.0%)	
False lumen	8 (32.0%)	7 (28.6%)	
Right renal artery, *n* (%)	25	25	1.000
True lumen	15 (60.0%)	15 (60.0%)	
Both lumens	3 (12.0%)	3 (12.0%)	
False lumen	7 (28.0%)	7 (28.0%)	

## Discussion

A total of 29 patients underwent TEVAR for TBAD following the conservative treatment of 13.9 ± 7.4 days since hospital admission. What is the optimal time window for TEVAR in the context of TBAD? TEVAR during the subacute phase of dissection (15–90 days since symptom onset) was associated with lower rates of postoperative adverse events compared with those during the acute phase (9). Of note, aortic rupture, retrograde type A dissection (RTAD), and disabling stroke were observed only in TEVAR during the acute phase (10). This elevated that the periprocedural risk is related to aortic fragility during the acute phase, making patients more vulnerable to mechanical complications such as aortic rupture or RTAD. Aortic remodeling was analyzed by evaluating the thrombus status of FL and aortic diameters at the thoracic aorta or the abdominal aorta level following TEVAR. TEVAR during the subacute phase of dissection yields better aortic remodeling compared with delayed intervention during the chronic phase (>90 days) (11). This is mostly attributed to the dissection flap becoming less compliant over time (11). The subacute phase is the optimal timing for TEVAR in TBAD to decrease the risk of intervention and achieve better long-term outcomes (12, 13).

There is a uniform consensus that LSA revascularization is the most suitable method for TBAD with insufficient proximal landing zone. The current mainstream ways for LSA revascularization consist of carotid-subclavian bypass or transposition and the chimney and fenestration techniques. Carotid-subclavian bypass or transposition is an effective technique for open surgical revascularization of the LSA. Long-term patency was excellent with a 5-year primary rate of 97%–98% for carotid-subclavian bypass and 100% for carotid-subclavian transposition (14, 15). However, surgical bypass is more invasive and has associated complications, which is not recommended for high-risk patients with advanced age or low cardiac function. The short-term complication rate, which was attributed to the carotid-subclavian procedures, was 29% (14). Perioperative complications included injury to the brachial plexus and phrenic nerve, neck hematoma, and chyle leak from the thoracic duct (14, 15). The ipsilateral stroke rate was reported to be 3.4% in 58 patients following LSA transposition over a mean follow-up of 2.8 years (16).

The introduction of the chimney or fenestration techniques makes it possible to preserve LSA blood flow with a percutaneous, totally endovascular approach. The chimney technique is achieved by the deployment of a covered or bare-metal LSA stent parallel to the aortic stent graft. The gutter between the chimney stent and the main aortic endograft carries a high risk of endoleak when using the chimney technique. The immediate overall rate of endoleak was reported at 15.3% after TEVAR combined with the chimney technique (17). Another critical issue for the chimney technique is the stent collapse or occlusion due to the radical force of the aortic stent graft on the chimney stent (17, 18). A meta-analysis involving 12 studies and 379 patients demonstrated that the perioperative rate of patency was 21% following the use of the chimney technique for treating aortic arch pathologies (19). For fenestrated devices, proper orientation of the fenestration will facilitate the alignment of the aortic endograft to the supra-aortic target arteries. Orientation of the fenestration toward the ostium of the target arteries in the angulated arch is difficult and risky due to the curve of the aortic arch (20). Precise deployment of the fenestrated aortic endograft is critical to correctly orient the fenestration toward the target arteries. We experienced the failure of aligning the fenestration into the LSA ostium and thus resorted to chimney endograft as a salvage. The currently available techniques for *in situ* fenestration are needle and laser punctures. *In situ* fenestration of the LSA is often challenging secondary to the tortuous anatomy of the proximal subclavian artery (21). Moreover, *in situ* fenestration requires a vertical upward angle of the target branch to the aortic arch (22). Tortuosity and the acute angle of the branch vessels greatly increase the procedural technical difficulty and complexity (23). Balloons for dilation of the fenestration might lead to fabric fraying and tearing, which adversely affects long-term stent-graft stability and fenestration durability (24). Metal ring stability and fabric material durability of the fenestrated endograft will need to be monitored closely by long-term radiologic follow-up examinations (25).

This clinical analysis reported our experience with the Castor unibody single-branched stent graft for TEVAR in 29 patients with TBAD involving the LAS. The initial technical success rate was 96.6% (28 of 29). There was no perioperative mortality, paraplegia and stroke did not occur, and no stent migration was observed. All the LSA branches remained patent during follow-up. Type Ⅰ endoleak between the aortic stent graft and the aorta was minute in one patient during follow-up and subsequently resolved after conservative therapy. One new entry tear occurred on the aortic root of the proximal edge of the stent graft, progressed to retrograde type A aortic dissection, and was treated open-surgically at 12 months after TEVAR. Four new entry tears were identified on the abdominal aorta distal to the stent graft and were observed conservatively. We can conclude that the unibody single-branched stent graft is feasible and offers encouraging results in the short term. A multicenter prospective trial reported 73 patients with TBAD, who were treated with the Castor stent graft, from 11 Chinese tertiary hospitals (26). The technique success rate was 97% (71 of 73), the intraoperative endoleak rate was 5% (4 of 73), the aorta-related mortality was 0% within 6 years, and the follow-up patency rate of the branch section was 93% (63 of 68) (26). A retrospective analysis in a single center reported 52 patients with TBAD receiving the Castor stent graft with an average follow-up of 16.8 months (27). The technique success rate was 100%, the patency rate of the branched segment was 100%, and there were no deaths and complications such as stent displacement, stenosis, fracture, occlusion, and type Ⅰ endoleak (27). These preliminary results justify the safety and efficiency of the Castor single-branched stent graft in the treatment of TBAD requiring LAS revascularization.

In the present study, we analyzed CT morphologic outcomes after Castor stent-graft implantation for TBAD. TEVAR with the Castor device effectively induced favorable aortic remodeling, which was observed as TL enlargement and FL thrombosis/obliteration at the thoracic aorta. We observed 33.3% of abdominal TL growth and 66.7% of abdominal TL stabilization or shrinkage after TEVAR with the Castor stent graft. The initial TL ratio at iliac bifurcation ≤21.0% was a reliable predictor for abdominal TL growth at follow-up. To the best of our knowledge, this is the first clinical study to evaluate abdominal TL enlargement and its pre-procedural CT predictors. The abdominal aorta bifurcates into the right and left common iliac arteries. The left and right common iliac arteries are the terminal branches of the abdominal aorta. TL growth at the abdominal aorta implies that the common iliac artery has an increased TL perfusion, which improves blood flow to the lower limb and relieves ischemic symptoms. The TL ratio in the short-axis view reflects the difference in hydrodynamic pressure and wall shear stress between TL and FL. A wide tear and an elevated FL pressure favor a reduced TL ratio especially at iliac bifurcation. TEVAR seals the primary entry tear, restores the TL bloodstream, and influences FL status. When the TL ratio at iliac bifurcation is ≤21%, TEVAR mitigates the pre-existing pulsatile pressure within the FL and repositions the intimal flap toward FL at the abdominal aorta.

Abdominal aortic perfusion patterns remained largely unchanged after utilization of the Castor stent graft in TBAD. Aortic remodeling and abdominal aortic branch perfusion have been largely described in patients with TBAD after standard TEVAR. TL regression combined with FL thrombosis and shrinkage is mostly limited to the thoracic aorta due to stent-graft coverage of the primary intimal tear (28–30). The dissected abdominal aorta appeared to be more prone to expansion over time, and growth at the abdominal aorta following standard TEVAR was mainly attributed to patent FL expansion (28–30). Although TEVAR seals the proximal tear and re-channels blood flow into TL while depressurizing the FL, this hemodynamic alteration does not impact perfusion patterns of abdominal aortic branches (31–33).

Three limitations existed in this study. First, the sample size was relatively small, with a total of 29 patients enrolled in this clinical study at a single department. Second, the follow-up period was relatively short. A larger sample size with a longer follow-up period is needed to better understand the long-term safety and efficacy of the Castor device. Third, abdominal branch perfusion was evaluated by vessel origin from TL, FL, or BL. The blood flow of the branch vessel was not qualified and compared for pre-TEVAR vs. the most recent follow-up.

## Conclusions

In patients with TBAD requiring a proximal landing zone to the LSA origin, the Castor unibody single-branched stent graft can offer an effective therapeutic strategy for retaining the LSA. TEVAR with the Castor stent graft successfully induces favorable aortic remodeling at the thoracic aorta, which is manifested as FL thrombosis and regression, TL expansion, and transaortic stabilization. Distal to the stent graft, the abdominal aorta might experience TL growth, TL shrinkage, or no change following TEVAR with the Castor stent graft. The initial TL ratio at aortic bifurcation of ≤20.1% is an independent predictor for TEVAR-induced TL growth. Perfusion patterns of abdominal aortic branches remain largely stable after TEVAR with the Castor stent graft.

## Data Availability

The raw data supporting the conclusions of this article will be made available by the authors, without undue reservation.
